# Elucidating the function of the prion protein

**DOI:** 10.1371/journal.ppat.1006458

**Published:** 2017-08-31

**Authors:** Giuseppe Legname

**Affiliations:** Scuola Internazionale Superiore di Studi Avanzati, Trieste, Italy; Washington University School of Medicine, UNITED STATES

The prion protein (PrP) has been extensively studied because of its central role in a group of neurodegenerative conditions collectively known as prion diseases. While a wealth of information is available for the pathology and transmission of these diseases, the molecular mechanisms involved are not yet clearly defined.

So, how do we learn about the molecular mechanisms underlying the pathogenic role of the PrP in disease? What have we learned about the physiological role of the PrP? Defining PrP function may shed light on pathological processes involved in prion diseases. The PrP has been shown to participate in several biological processes, including neuritogenesis, neuronal homeostasis, cell signalling, cell adhesion, and a protective role against stress. This pleiotropism has led to confusion about the precise molecular function(s) of the PrP. This essay shall attempt to clarify the most relevant physiological roles of the protein in the context of the central and peripheral nervous system.

## The PrP

The PrP can exist in 2 distinct conformations: the host-encoded, physiological cellular prion protein (PrP^C^) and the pathogenic isoform denoted as prion (usually referred to as PrP^Sc^). The latter plays a key role in the pathological outcome of prion diseases, while the former is a ubiquitous protein expressed in most cell types in mammals.

The PrP^C^ is encoded by the *Prnp* gene located on chromosome 20 in humans (*PRNP*) and chromosome 2 in mice. Depending on the species considered, the *Prnp* gene contains either 2 or 3 exons, with the entire coding region being contained in the last exon, thus excluding possible alternative splicing [1].

The murine PrP^C^ is a protein of about 254 amino acids prior to post-translational modifications and in its mature form is a 208–amino acid polypeptide, which is glycosylphosphatidylinositol (GPI) anchored to the outer leaflet of the cellular membrane with a unique primary sequence.

The unstructured N-terminal domain possesses distinctive sequences identified as octarepeats, solely represented in PrP^C^, which are unique among all proteins. These octarepeat regions, with a consensus sequence of PHGGGWGQ, contain hystidine residues able to bind monovalent and divalent cations, such as copper ions Cu^+^ and Cu^2+^. The octarepeat sequence of the PrP binds Cu^2+^ with distinct coordination modes [2].

**Fig 1:**
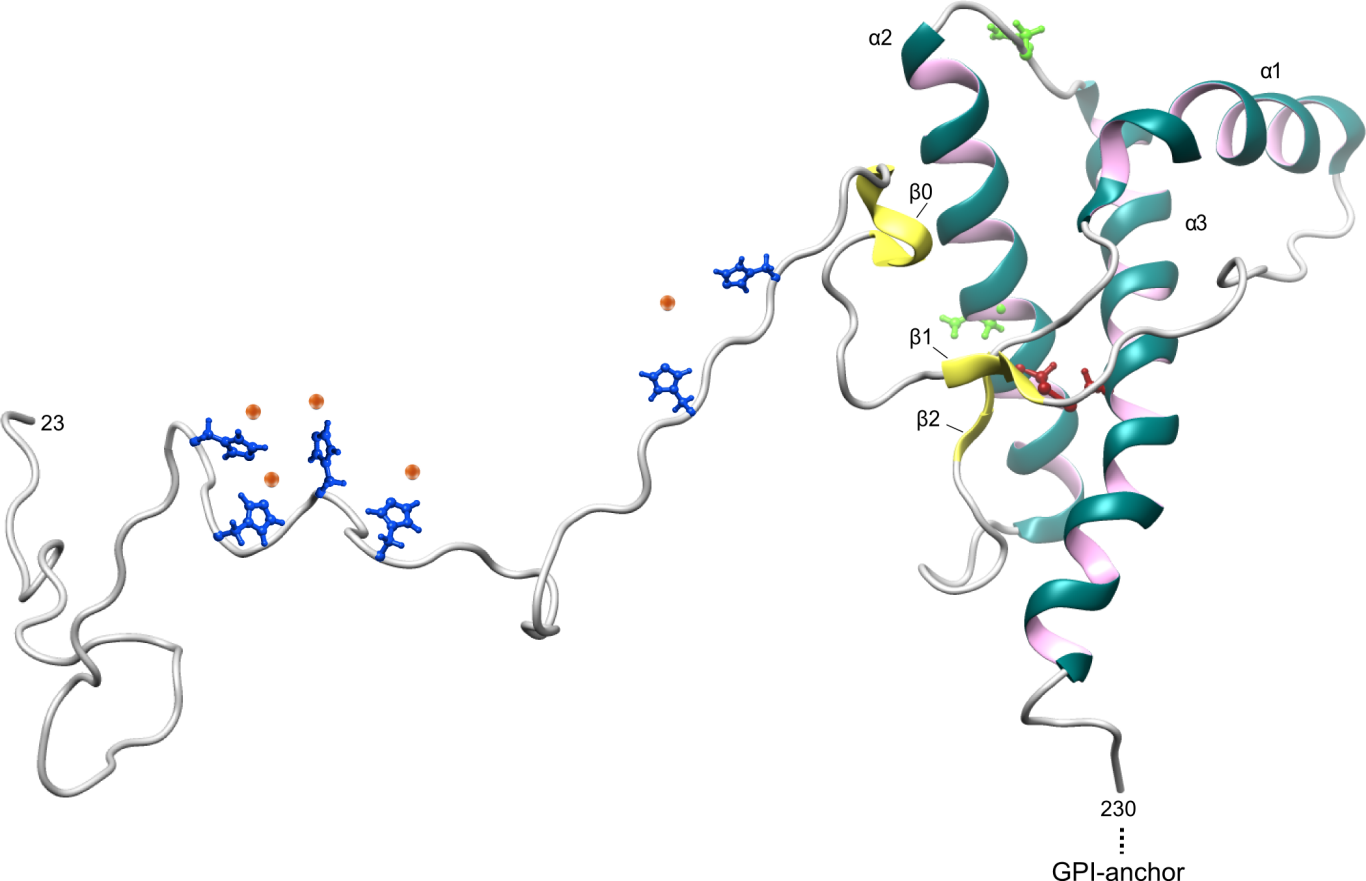
Schematic representation of cellular prion protein (PrP^C^). The N-terminal domain of PrP^C^ is unstructured and possesses distinctive sequences identified as octapeptide repeats (see main text for details). These octarepeat regions contain hystidine residues (in blue) able to bind monovalent and divalent cations, such as copper ions Cu^+^ and Cu^2+^ (orange dots). The C-terminus contains a single disulphide bridge (in red) and 2 glycosylation sites. The asparagine residues involved in the glycosylation of the protein are represented in green. The overall structure of the C-terminus is composed of 2 short antiparallel beta sheet strands, namely β1 and β2 (in yellow) and 3 alpha helices, indicated as α1, α2, and α3. A third beta sheet strand has been recently identified and named β0 (in yellow).

On the other end, the protein presents a well-structured C-terminal domain structurally conserved in all mammals. The C-terminus contains a single disulphide bridge and 2 glycosylation sites. The asparagine residues involved in the glycosylation of the protein provide the presence of 4 different isoforms of the protein; they could be both occupied by glycans or, alternatively, only 1 could be glycosylated or none at all. The overall structure of the C-terminus is composed of 2 short antiparallel beta sheet strands and 3 alpha helices, which provide a compact conformation retained in all mammalian species. More recently, a third beta sheet strand has been identified, which may play a role in prion conversion (Fig 1) [3].

Via additional post-translation modifications, PrP^C^ may be subjected to proteolytic processing. One of the cleavage sites is present in the central region of the protein and produces the N-terminal N1 soluble fragment and the GPI-anchored C-terminal C1 fragment. PrP^C^ may also be present as a soluble full-length isoform, resulting either from a phospholipase cleavage of the GPI anchor or from proteolytic processing at the C-terminus. These varieties of post-translational modifications can give rise to several different isoforms and this could hamper efforts on defining PrP^C^ function(s) [4].

## Lessons learned from knockout mouse models

With the exception of 3 PrP knockout mouse models, in which ectopic expression in the central nervous system of the PrP paralogue Doppel leads to loss of Purkinje cells in the cerebellum, the evidence that most mouse models’ knockout for the PrP do not show gross abnormalities indicates that PrP^C^ may be dispensable for embryonic development and adulthood. Nevertheless, several mouse models in which the *Prnp* gene is disrupted have been developed [5]. Early studies using these models have implicated PrP^C^ in circadian rhythms and sleep dysfunctions [6], altered olfactory behavior [7], neuritogenesis and neural stem/precursor cells differentiation in the central nervous system [8], and myelination of neurons in the peripheral nervous system [9].

In addition, more recent work has characterized PrP^C^ involvement in synaptic plasticity and N-methyl-d-aspartate receptors (NMDARs) regulation [10, 11].

Overall, these knockout models have been instrumental for defining PrP^C^ function and despite their limitations, they are still used in characterizing PrP^C^ physiological roles in the central and peripheral nervous system.

## The function(s) of the PrP

One of the most intriguing function(s) of PrP^C^ is its involvement in cell signalling. Because of its extracellular localization, the protein could mediate environmental molecular signals to the cell. Transduction of the signals cannot be mediated directly by PrP^C^ because it is GPI anchored to the cellular membrane without direct access to the cytosol but would require interactions with other transmembrane proteins.

Perhaps the most important study and the first evidence that PrP^C^ may be involved in mediating extracellular signals is the description of a caveolin-1-dependent coupling of PrP^C^ to the proto-oncogene tyrosine-protein kinase Fyn (Fyn) [12]. Since this seminal work, it became clear that PrP^C^ could exert its function by partnering with other membrane proteins to convey cellular signalling. The neural cell adhesion molecule (NCAM) was identified as one preferential interactor of PrP^C^ [13]. Through physical interaction with NCAM, PrP^C^ can promote neuritogenesis via the tyrosine kinase Fyn [14–17]. In this work, the N-terminal domain of PrP^C^ is essential for regulating the neurite outgrowth and guidance function, indicating that the N-terminus of the protein, which includes the octarepeats region, is essential for its function [17]. Notably, a soluble form of full-length PrP^C^ has been used for focal stimulation of neurite outgrowth and guidance [17].

In addition, in another work it has been shown that PrP^C^ plays a critical role in NCAM-dependent neuronal differentiation of neural stem/precursor cells [18].

PrP^C^ is developmentally regulated and its high expression in the immature brain could be relevant in regulating neurogenesis and cell proliferation [19]. A recent study shows that PrP^C^ plays a crucial role in regulating via protein kinase A (PKA) synaptic plasticity in the developing hippocampus, therefore contributing to proper synaptic formation in adulthood [10].

An important function linked to PrP^C^ expression is its involvement in myelin formation and maintenance. Aging PrP knockout mice present a clear phenotype in which the peripheral nervous system shows demyelinating disease [9]. Molecular studies have shown that the N-terminus of PrP^C^ acts as an agonistic ligand of the adhesion G-protein coupled receptor G6 (Adgrg6) receptor, the function of which is critical for myelin maintenance [20].

## PrP regulates NMDAR

One of the most detailed functional studies recently published deals with the involvement of the cellular form of the prion protein PrP^C^ and copper ions in NMDAR S-nitrosylation and activity. By exploiting PrP knockout mice, the authors showed that the depletion of PrP^C^ is associated with a reduction in the S-nitrosylation of the 2 NMDAR subunits GluN2A and GluN1, while not affecting the levels of the corresponding proteins at the synapse. The sensitivity of PrP knockout versus wild-type organotypic hippocampal cultures to N-methyl-d-aspartate (NMDA)-mediated excitotoxicity was monitored under a great variety of conditions that were selected in order to assess the involvement of calcium, copper, nitric oxide (NO), NMDA, α-amino-3-hydroxy-5-methyl-4-isoxazolepropionic acid (AMPA)/kainite, or GluN2B receptors. These experiments unambiguously highlight a higher sensitivity of PrP knockout mouse cultures to NMDA-mediated excitotoxicity, which can be reversed upon exposure to the NO donor S-nitrosoglutathione (GSNO). Conversely, the results substantiate an increased sensitivity of wild-type cultures to NMDA-dependent excitotoxicity when copper or NO is chelated.

**Fig 2:**
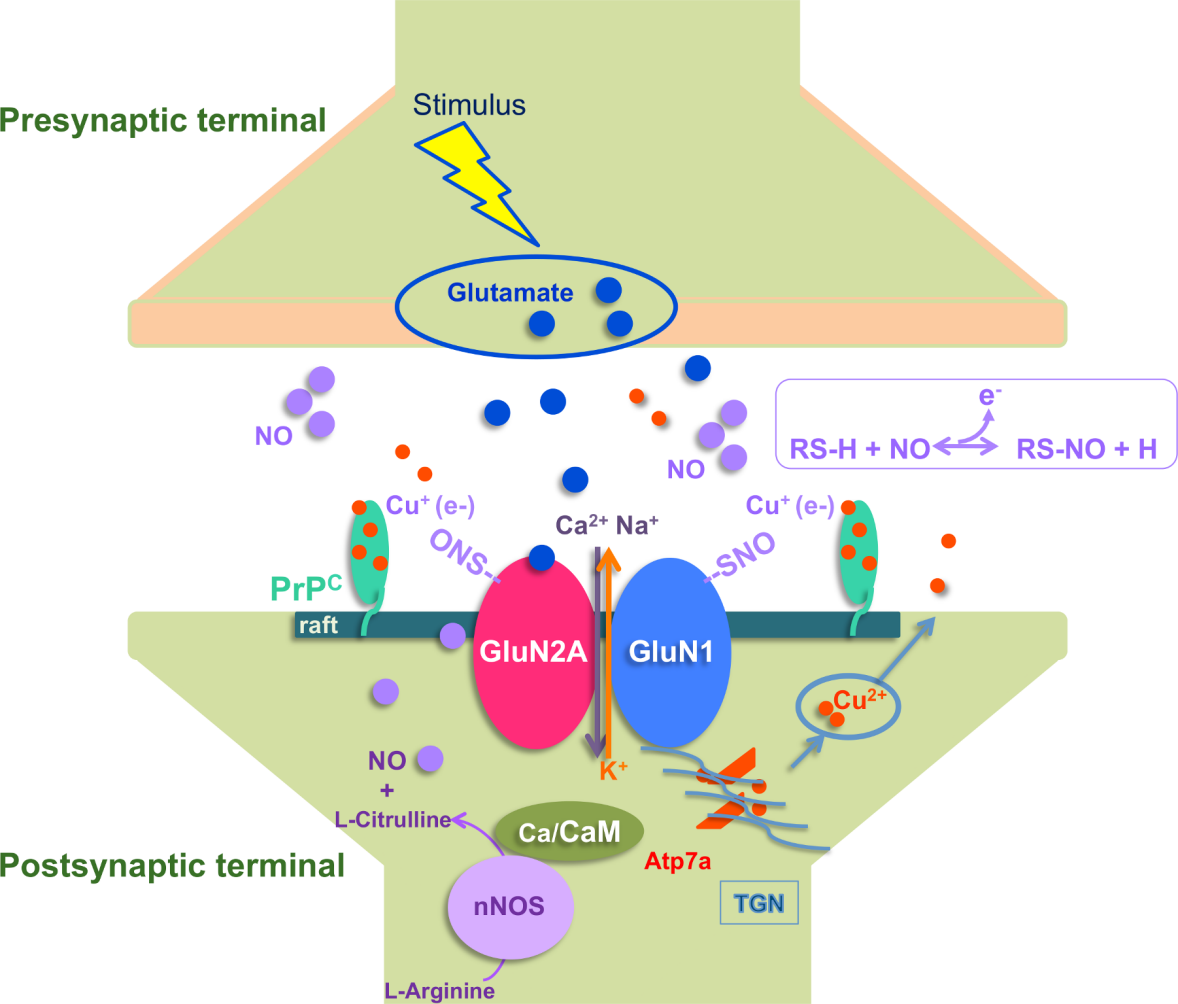
Schematic representation of the mechanism of cellular prion protein (PrP^C^)-mediated S-nitrosylation of N-methyl-d-aspartate receptor (NMDAR). One mechanism controlling NMDAR as well as other membrane ion channels involves direct modulation by nitric oxide (NO). Catalytic amounts of copper can act as electron acceptors promoting the reaction of NO with thiols, providing inhibitory S-nitrosylation (RSNO) of NMDAR. The RSNO formation can take place only after one-electron oxidation from the free radical NO to NO^+^ by transition metal. In brief, glutamate is released from the presynaptic terminal of neurons and activates NMDAR on the postsynaptic terminal. NMDAR activation and opening generates Na^+^ and Ca^2+^ influx and K^+^ efflux. In the cytosol, upon entrance, Ca^2+^ ions bind to different proteins, among these, calmodulin (CaM). The CaM bound to Ca^2+^ triggers neuronal nitric oxide synthase (nNOS) and copper-transporting ATPase 1 (Atp7a). Activation of nNOS leads to NO release in the synaptic cleft. Activation of Atp7a in the trans-Golgi network (TGN) ensues in Cu^2+^ release in the synaptic space. Transient free Cu^2+^ ions are immediately bound by copper-binding proteins like PrP^C^, which is highly expressed in both pre- and postsynaptic terminals. PrP^C^ has high affinity for both Cu^2+^ and Cu^+^ and can be found in lipid raft domains, which also contain NMDAR. NO can react with extracellular cysteine thiols of NMDAR subunits GluN1 and GluN2A, leading to cysteine S-nitrosylation (SNO-Cys). The S-nitrosylation inhibits NMDAR activation by closing the channel. The chemical reaction between NO and cysteine thiol requires the presence of an electron acceptor such as Cu^2+^. PrP^C^ coordinates Cu^2+^ ions, which support the reaction of NO with thiols, leading to the S-nitrosylation of GluN1 and GluN2A and therefore NMDAR inhibition.

The molecular mechanism by which PrP^C^ acts to regulate NMDAR S-nitrosylation regulation can be summarized as follows. Upon glutamate release from the presynaptic terminal, NMDARs are activated on the postsynaptic terminal, leading to calcium entry. Via a series of molecular mechanisms, NO and copper ions are released in the synaptic cleft. Released Cu^2+^ ions are rapidly bound by copper-binding proteins including PrP^C^, which is highly expressed in both presynaptic and postsynaptic terminals. PrP^C^ has high affinity for both Cu^2+^ and Cu^+^ and it may reside in lipid raft domains, which also contain NMDAR. Synaptic NO can react with extracellular cysteine thiols of NMDAR subunits GluN1 and GluN2A, leading to cysteine S-nitrosylation. The S-nitrosylation inhibits NMDAR activation by closing the channel. The chemical reaction between NO and cysteine thiol requires the presence of an electron acceptor such as Cu^2+^. According to this model, PrP^C^ positions Cu^2+^ ions that support the reaction of NO with thiols, leading to the S-nitrosylation of GluN1 and GluN2A, thus inhibiting NMDAR [11, 21] (Fig 2).

## Future directions

In recent years, several function(s) of PrP^C^ have been identified. The use of PrP knockout mouse models has been influential for studying and clarifying the molecular mechanisms in which the protein is involved. By learning the physiological function(s) of PrP^C^, our understanding of the neuropathological processes underlying prion diseases may progress towards the development of novel therapeutic approaches to such devastating disorders [22].
